# Enhanced neprilysin-mediated degradation of hippocampal Aβ42 with a somatostatin peptide that enters the brain

**DOI:** 10.7150/thno.50263

**Published:** 2021-01-01

**Authors:** Fadi Rofo, Canan Ugur Yilmaz, Nicole Metzendorf, Tobias Gustavsson, Chiara Beretta, Anna Erlandsson, Dag Sehlin, Stina Syvänen, Per Nilsson, Greta Hultqvist

**Affiliations:** 1 Department of Pharmaceutical Biosciences, Uppsala University, Uppsala, Sweden; 2 Department of Public Health and Caring Sciences, Uppsala University, Uppsala, Sweden; 3 Department of Neurobiology, Care Sciences and Society, Karolinska Institutet, Stockholm, Sweden

**Keywords:** Amyloid-β (Aβ) / blood-brain barrier (BBB) / neprilysin / somatostatin (SST) / transferrin receptor (TfR)

## Abstract

**Background:** Aggregation of the amyloid-beta (Aβ) peptide is one of the main neuropathological events in Alzheimer's disease (AD). Neprilysin is the major enzyme degrading Aβ, with its activity enhanced by the neuropeptide somatostatin (SST). SST levels are decreased in the brains of AD patients. The poor delivery of SST over the blood-brain barrier (BBB) and its extremely short half-life of only 3 min limit its therapeutic significance.

**Methods:** We recombinantly fused SST to a BBB transporter binding to the transferrin receptor. Using primary neuronal cultures and neuroblastoma cell lines, the ability of the formed fusion protein to activate neprilysin was studied. SST-scFv8D3 was administered to mice overexpressing the Aβ-precursor protein (AβPP) with the Swedish mutation (APPswe) as a single injection or as a course of three injections over a 72 h period. Levels of neprilysin and Aβ were quantified using an Enzyme-linked immunosorbent assay (ELISA). Distribution of SST-scFv8D3 in the brain, blood and peripheral organs was studied by radiolabeling with iodine-125.

**Results:** The construct, SST-scFv8D3, exhibited 120 times longer half-life than SST alone, reached the brain in high amounts when injected intravenously and significantly increased the brain concentration of neprilysin in APPswe mice. A significant decrease in the levels of membrane-bound Aβ42 was detected in the hippocampus and the adjacent cortical area after only three injections.

**Conclusion:** With intravenous injections of our BBB permeable SST peptide, we were able to significantly increase the levels neprilysin, an effect that was followed by a significant and selective degradation of membrane-bound Aβ42 in the hippocampus. Being that membrane-bound Aβ triggers neuronal toxicity and the hippocampus is the central brain area in the progression of AD, the study has illuminated a new potential treatment paradigm with a promising safety profile targeting only the disease affected areas.

## Introduction

Alzheimer's disease (AD) is a multifactorial neurodegenerative disorder affecting 40 million people worldwide 1. Its prevalence rises with age, which is the main risk factor 2. The two main neuropathological events in AD are extracellular aggregation of amyloid-beta peptide (Aβ) and intracellular deposition of hyperphosphorylated tau. The Aβ peptide is produced by enzymatic digestion of the type-1 membrane protein amyloid-precursor protein (APP). The cleavage of APP results in Aβ peptides of different lengths, but peptides of 40 and 42 amino acids long are the most common 3. Aggregated Aβ peptides can self-propagate the aggregation of other Aβ species. The aggregates can be of many different sizes, including soluble intermediate species (oligomers and protofibrils) and insoluble fibrils, which constitute the amyloid plaques. The Aβ42 peptide is more hydrophobic, making it more prone to aggregate, which in turn makes it more neurotoxic than the Aβ40 peptide 4. Pathological accumulation of aggregated Aβ is associated with neuronal toxicity and synaptic dysfunction, eventually leading to neuronal death 5. A number of previous studies indicate that the intermediate soluble species are the most toxic forms of Aβ 6-9. Interaction of Aβ with the membrane of the neurons can induce cell toxicity and affect its aggregation cascade 10,11. These point towards the significance of targeting membrane-bound Aβ over the soluble one.

Immunotherapy against Aβ is a promising therapeutic strategy in the fight against AD. Several monoclonal antibodies are currently in clinical trials targeting different species of Aβ 12, focusing on halting the pathological aggregation of Aβ or removal of Aβ aggregates. Due to the design of monoclonal antibodies, they only target rather large aggregates like protofibrils and mature fibrils 13, meaning that small aggregates like oligomers will not be affected by these treatments. Since these antibodies poorly pass the blood-brain barrier (BBB), it is also argued that they mainly target aggregates that are located at the hotspots where these antibodies can reach like the cerebrospinal fluid (CSF) 14-18. These drugs have had very limited effects on cognition in clinical trials 13,19. Another potential therapeutic intervention in AD is to enhance the degradation and clearance of Aβ prior to the formation of aggregates, which has the potential to have a larger effect on nerve health and cognition than the current therapies in clinical trials. This can be achieved by both increasing the expression and/or activity of the Aβ degrading enzymes, including neprilysin 20, and by reaching aggregates at new locations.

Neprilysin is the major Aβ degrading enzyme 21. It is a 90-110 kDa membrane bound metallopeptidase protein, localized mainly at the pre-synaptic terminals 22. Lower levels of neprilysin have been detected in postmortem brain tissue from AD patients compared to non-AD brains 23. Importantly, high expression of the naturally occurring neprilysin is associated with a decrease in the accumulation of Aβ in transgenic mouse models of AD 20,24. In addition, increased levels of Aβ have been observed in neprilysin knock-out mice 25,26, whilst gene therapy approaches with neprilysin lower Aβ pathology and improve cognition in AD mouse models 27.

Neprilysin activity can be increased by the neuropeptide somatostatin (SST) 28, a cyclic peptide with either 14 or 28 amino acids, produced by the neuroendocrine cells both in the brain and the periphery 29. In the central nervous system, SST is involved in modulating neurotransmission and neuroplasticity 30, exerting its physiological effects through interaction with a family of five G-protein coupled somatostatin receptors (SSTR) 31. Among these, the subtypes SSTR1 and SSTR4 are highly expressed in the hippocampus and neocortex 32,33. The levels of SST decrease in the brain and cerebrospinal fluid (CSF) with age and in patients with AD, potentially as an effect of the loss of somatostatinergic interneurons 34,35. This decline is considered to be one of the earliest biochemical changes in the AD brain, which also include abnormalities in the SSTR expression and density 36.

One of the main challenges in AD immunotherapy is the limited passage of protein drugs across the BBB 37,38. One of the mechanisms that could facilitate the delivery of large molecules, such as protein- and antibody-based drugs, across the BBB is receptor-mediated transcytosis via the transferrin receptor (TfR) 39-41. Some of the antibodies that bind to TfR are able to pass the BBB, with one such example being the rat monoclonal antibody 8D3 that recognizes mouse TfR-1 42. Previously, we have designed a bispecific antibody using a single chain fragment variable (scFv) of the 8D3 antibody and recombinantly linked it to an antibody that selectively targets the soluble protofibrils of Aβ 43. Compared with the parent antibody without the transferrin binding moiety, this bispecific antibody exhibited an 80-fold and 10-fold increase in brain uptake when administered at tracer and therapeutic doses respectively 43. The antibody with the transporter co-localized very well with the pathological aggregates in transgenic AD mice, which was not the case for the antibody without the transporter 17. To date, the BBB transporter scFv8D3 has been fused to antibodies or fragments of antibodies for use as diagnostic and therapeutic tools in AD 17,44.

SST could potentially be used as a therapeutic option in AD through its ability to enhance the activity of neprilysin and thereby reducing the amount of Aβ in the brain. Previous studies have tested small molecule drug agonists of SSTR4 45,46, which activated neprilysin and decreased Aβ. In the current study, we instead use the specific naturally occurring 14 amino acid SST peptide as a therapeutic option to reduce the Aβ brain concentrations through neprilysin activation. However, the peptide is too large (around 1.7 kDa) to pass the BBB on its own when peripherally administered. By recombinantly linking scFv8D3 to the SST peptide, followed by intravenous injection of the formed bispecific fusion protein, we could facilitate the brain delivery of SST. This design enhanced the levels of neprilysin in the hippocampus, which led to reduced levels of membrane bound Aβ42.

## Results

### Generation of recombinant SST-scFv8D3 and characterization of its binding to TfR

**Figure 1:**
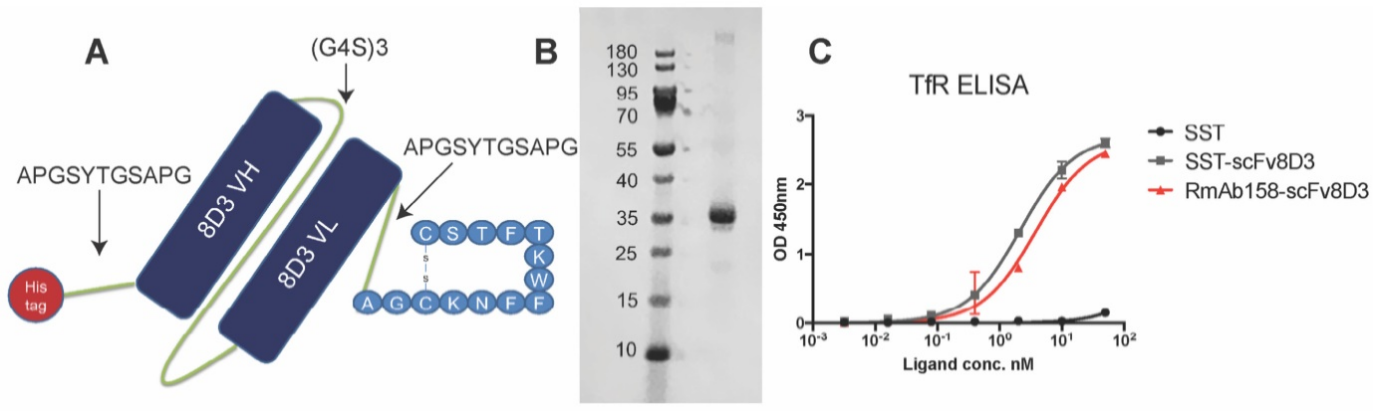
** Design of SST-scFv8D3 and characterisation of its purity and binding properties to transferrin receptor TfR.** (A). Schematic picture of SST-scFv8D3, where scFv of 8D3 is added to the N-terminal end of SST. A poly-6 His-tag is added to the N-terminal end of scFv8D3 for purification purposes. (B). SDS-PAGE of the purified SST-scFv8D3 showing a single band at the expected size, approximately 31 kDa. (C). ELISA displaying similar binding to TfR between SST-scFv8D3 and RmAb158-scFv8D3, using SST (without scFv8D3) as a negative control.

The previously used BBB transporter scFv8D3 17,43,44 was recombinantly fused to the N-terminal end of the 14 amino acid SST peptide through an in-house designed linker (APGSYTGSAPG) 43 with a poly-6 His-tag on the N-terminal end of the construct (Figure 1A). To ensure that no alpha helices are formed in the linker, a proline was added to the beginning and end. Serine, threonine and glycine were added to the linker to provide flexibility and hydrophilicity, with the addition of tyrosine providing potential labelling possibilities. We believed that a rather short linker would be sufficient due to the small size of the peptide. The molecular weight of the whole protein was around 31 kDa. The protein was produced by transient expression in Expi293 cells 47 with yields of around 30 mg per liter of transfected cell cultures. The construct was expressed in these mammalian cells since it was essential that a disulfide bond between the two cysteine residues in SST structure was formed, which is required for the biological activity 48. Non-cyclic SST peptide without the disulfide bond is also more prone to aggregate 49. SDS-PAGE analysis displayed a single band, at approximately 31 kDa, confirming the size and purity of the recombinant SST-scFv8D3 protein (Figure 1B). Using Image Studio software (version 5.2.5), we estimated the purity of our construct to be around 70% (Figure S1).

To assess efficient binding to the TfR, an indirect ELISA setup was performed 50 with RmAb158-scFv8D3 as a positive control, which has been shown to interact with TfR monovalently 43. SST-scFv8D3 and RmAb158-scFv8D3 demonstrated similar binding affinity to TfR, indicating a functional binding of SST-scFv8D3 (Figure 1C). Using a competition ELISA, the affinity of scFv8D3 to TfR, either alone or conjugated with a domain in an identical position as the SST peptide, has been previously determined to be approximately 24 nM 44.

### SST-scFv8D3 enhances cell surface neprilysin activity *in vitro*

**Figure 2:**
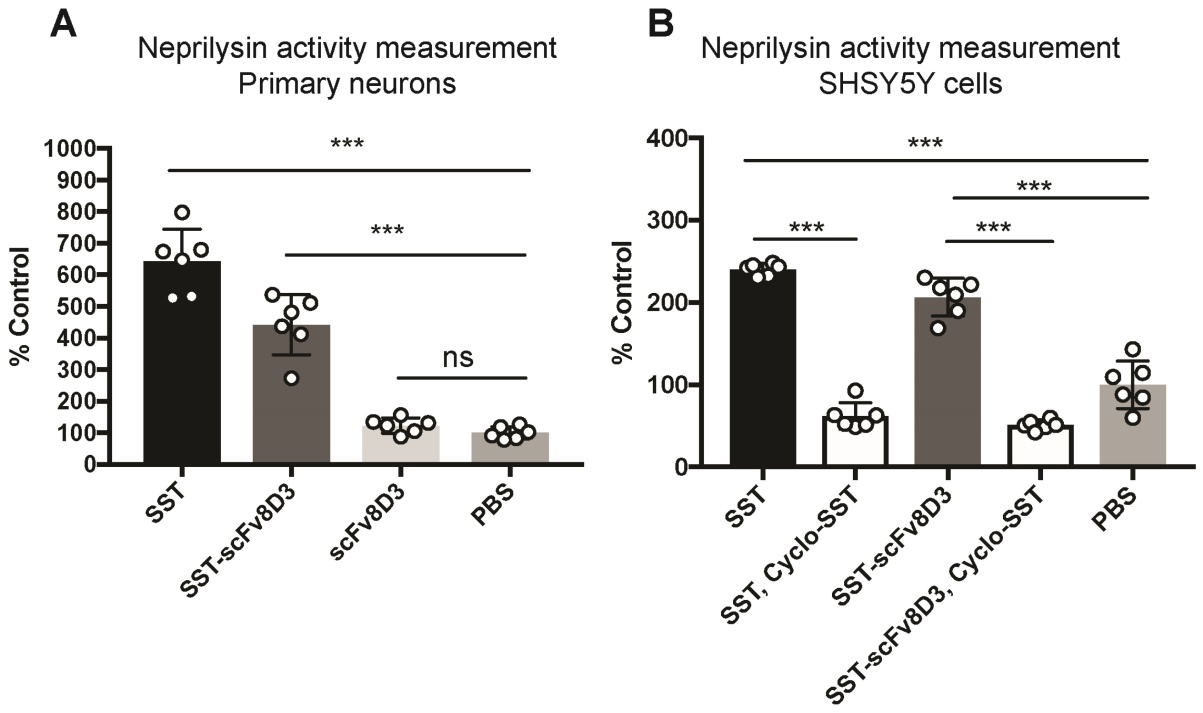
** Enhanced cell-surface activity of neprilysin by SST-scFv8D3 in primary neurons and SHSY5Y cells.** (A). Primary murine neurons incubated for 24 h with 1 μM of SST, SST-scFv8D3 and scFv8D3, using PBS as a negative control. The recombinant protein SST-scFv8D3 significantly enhances the activity of neprilysin when compared to scFv8D3 and PBS. (B). Human SHSY5Y cells incubated for 24 h with 10 μM of SST and SST-scFv8D3, with or without cyclo-SST, using PBS as a negative control. The recombinant protein SST-scFv8D3 significantly enhances the activity of neprilysin when compared to PBS. Co-administration of cyclo-SST abolished neprilysin activation. Results are presented as mean ±SD. One-way ANOVA was used followed by Dunnett's post hoc test. A significant p-value is defined as: >0.5 (ns), <0.05 (*), <0.01 (**), <0.005 (***).

Previous studies have demonstrated that Aβ degradation can be increased by enhancing the activity of membrane-bound neprilysin in primary neurons 51. First, we investigated whether the recombinantly produced SST-scFv8D3 protein could activate neprilysin *in vitro*, despite having the scFv8D3 attached to the N-terminal end of SST peptide (no free N-terminal). We followed a previously established protocol 52, using SST as a positive control and PBS as a negative control. Upon exposure to 1 μM of SST or SST-scFv8D3 for 24 h, neprilysin activity in primary neurons was significantly increased (Figure 2A). The measured activation of neprilysin by SST-scFv8D3 preserved around 70% of the activation obtained by SST alone (Figure 2). The purity of the SST-scFv8D3 protein was only 70% (Figure S1), which could account for the 30% difference in activity of neprilysin since the concentration used was based on total protein concentration in the sample. Taking this into account, SST-scFv8D3 could probably be considered equally as potent an activator of neprilysin as SST alone. The scFv8D3 alone did not show any effect on neprilysin activation (Figure 2A). Using the same method, we also measured neprilysin activity in SHSY5Y cells, which has been previously confirmed to express neprilysin 52. Since endogenous neprilysin activity is lower in SHSY5Y cells compared to primary neurons 52, a higher concentration of SST and SST-scFv8D3 (10 μM) was used. The same pattern of activation was also achieved in SHSY5Y cells (Figure 2B). Co-treatment of these cells with cyclo-SST, a non-selective SSTR antagonist, blocked the activation (Figure 2B). The scFv of 8D3 alone was not added in case of human SHSY5Y cells since it recognizes mouse TfR, which is not expressed on human neuroblastoma cells lines.

### Distribution of radiolabeled SST-scFv8D3 in the blood, brain, and peripheral organs

**Figure 3:**
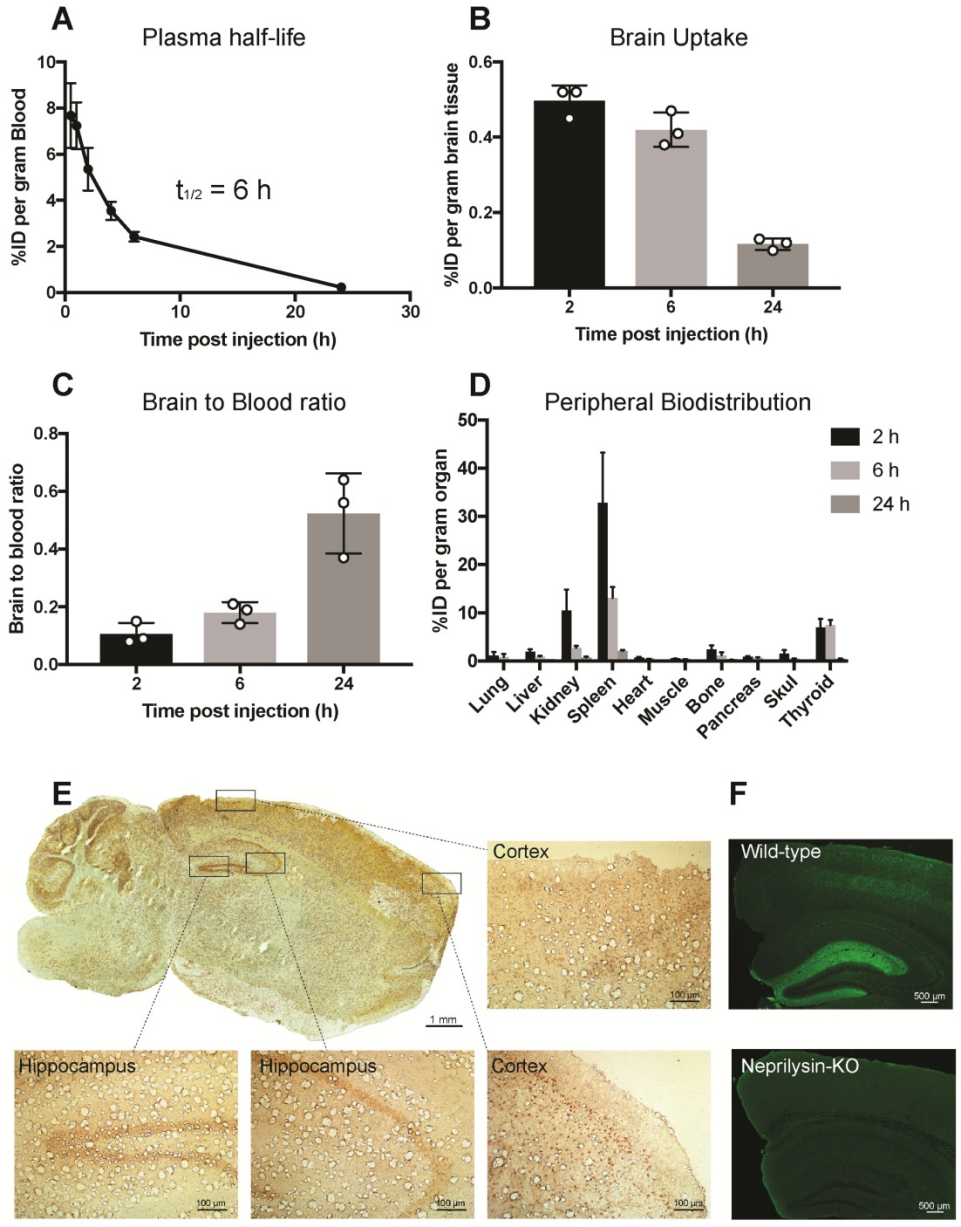
** Pharmacokinetics and distribution of iodine-125 labelled SST-scFv8D3 when given intravenously to 7-8 month old C57Bl/6 mice (n=3 per group).** (A). Plasma concentrations, expressed as percentage of injected dose (%ID) per gram blood of [^125^I]SST-scFv8D3. The plasma-half-life was estimated to 6 h, which is 120 times longer than the blood half-life of SST peptide alone. (B). Brain concentration, expressed as %ID per gram brain tissue, at 2, 6 and 24 h after intravenous injection of [^125^I]SST-scFv8D3. High brain uptakes observed at 2 and 6 h post injection which then declined at 24 h. (C). Brain-to-blood concentration ratio of SST-scFv8D3 at 2, 6 and 24 h post intravenous injection showing that the construct was retained in the brain longer than in the blood. (D). Concentration in the peripheral organs, expressed %ID per gram tissue. Spleen and kidney displayed high uptake 2 h post intravenous injection of [^125^I]SST-scFv8D3. (E). Regional distribution of SST-scFv8D3 in the brain 24 h post injection, detected with anti-His tag immunostaining (scale bar 1 mM). A high signal was mainly detected in the hippocampal area, cerebral cortex and the cerebellum. Boxed areas show the distribution of the drug in two regions of the cerebral cortex and the hippocampus (scale bar 100 μM). (F). Confocal images of neprilysin immunostaining in coronal sections prepared from the hippocampus of C57Bl/6 and neprilysin knock-out mice (scale bar 500 μM).

Pharmacokinetics and the ability of scFv8D3 to enhance SST peptide delivery into the brain *in vivo* was studied in 7-8 month C57Bl/6 wild-type mice. SST-scFv8D3 was labelled with iodine-125, which resulted in an activity of 0.24 MBq/μg (7.4 MBq/nmol). The plasma half-life of the recombinant protein, based on blood samples obtained at 0.5, 1, 2, 4, 6 and 24 h post injection, was around 6 h (Figure 3A). This is 120 times longer than the plasma half-life of intravenously injected SST peptide, which has been demonstrated to be only three min 48,53. Because of the short half-life of SST, it was not used as a control in this study. Brain concentration in mice injected intravenously with tracer doses (0.04 mg/kg) of [^125^I]SST-scFv8D3, presented as percentage of in injected dose (%ID) per gram brain tissue, at 2, 6 and 24 h post injection was 0.49±0.04, 0.42±0.04 and 0.11±0.01, respectively (Figure 3B). The brain-to-blood concentration ratio, which is based on the measure of the equilibrium across the BBB, was around 3 times higher 24 h post injection compared with the 2 and 6 h time points (Figure 3C). This indicates that [^125^I]SST-scFv8D3 is cleared from the blood faster than from the brain. Free iodine will not stay in the brain and hence the high brain signal indicates that our radiolabeled construct is stable during the time of analysis. A thorough analysis of the BBB transport of the shuttle in wild-type and transgenic mice with AD has been done earlier. The distribution of the intravenously injected recombinant protein in peripheral organs is shown in Figure 3D. Among the peripheral organs, the spleen and kidney displayed the highest concentrations. However, the concentration of the protein in these organs decreased drastically with time. The regional distribution of SST-scFv8D3 was investigated with immunostaining of the brain 24 h post injection. We detected a high signal in the hippocampal area, the cerebral cortex and the cerebellum (Figure 3E). The SSTR1-4 are highly expressed in the hippocampal area and the cerebral cortex 32. The high signal detected in the cerebellum is probably due to the rich blood supply in this region compared to the rest of the brain, resulting in the binding of SST-scFv8D3 to the SSTR3 present in this area. However, the cerebellum does not have a high expression of neprilysin and it is believed that SST does not regulate neprilysin activity in this area of the brain. Knocking out SST in the cerebellum does not alter neprilysin activity as it does in the hippocampus 28. Importantly, as shown with neprilysin immunostaining, the enzyme levels are relatively high in the hippocampus (Figure 3F) and co-localizes very well with the hippocampal distribution of SST-scFv8D3.

### Single intravenous injection of SST-scFv8D3 increases brain levels of neprilysin, but has no detectable effects on Aβ

**Figure 4:**
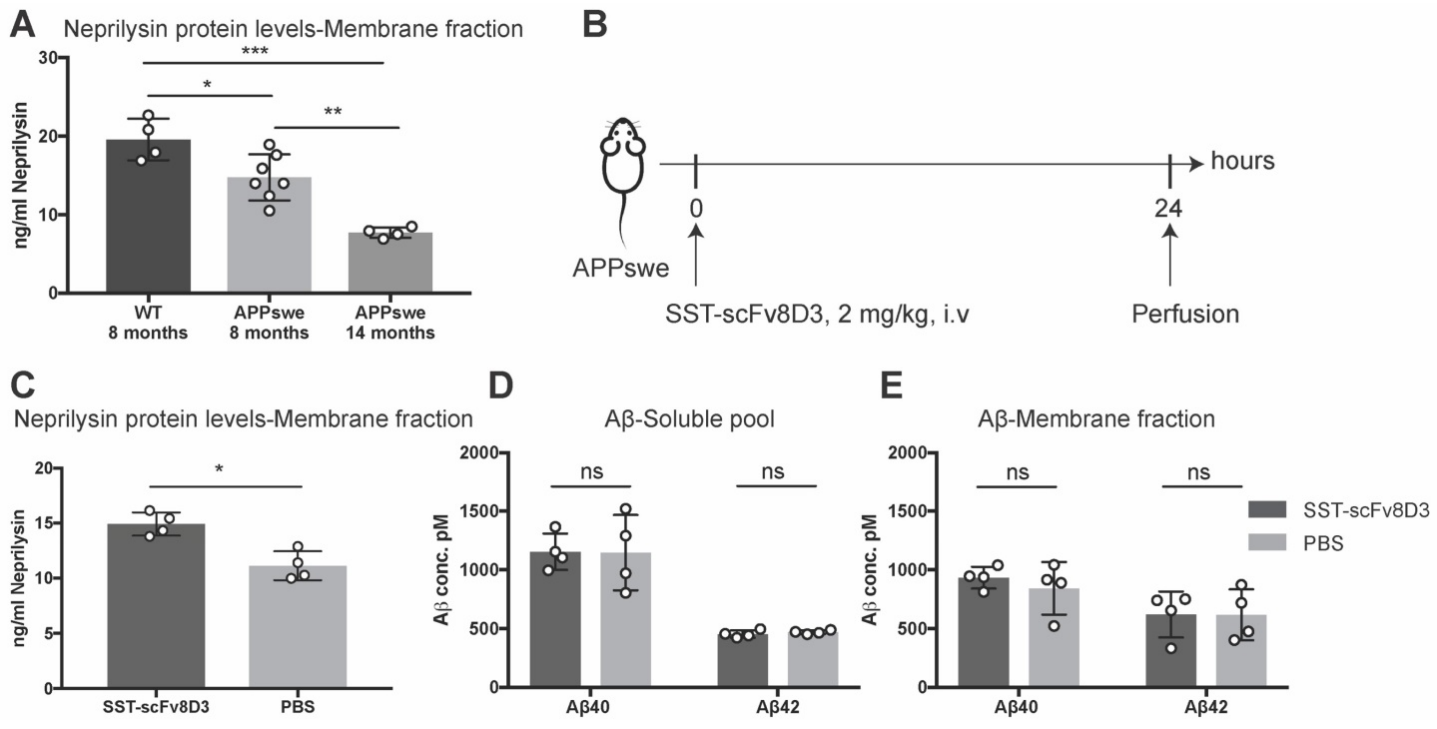
** Single intravenous injection of SST-scFv8D3 increases brain levels of neprilysin but has no detectable effect on Aβ levels.** (A). Comparison of neprilysin expression in the brain of wild-type mice (n=4), eight-months old APPswe (n=7) and fourteen-months old APPswe mice (n=4). APPswe mice exhibit a significant reduction in neprilysin levels compared to wild-type. A significant age-dependent decline in neprilysin protein levels is detected between the APPswe groups. (B). Schematic representation of the first therapeutic study with SST-scFv8D3. Fourteen-month old transgenic mice harbouring the Swedish mutation in APP (APPswe) injected intravenously with 2 mg/kg of SST-scFv8D3 (n=4) or PBS (n=4) as a negative control. Mice euthanized 24 h post injection by perfusion with physiological saline. (C). Significant increase in the levels of neprilysin in the membrane fraction (TBS-Triton soluble) of the brain of the treatment group. No differences detected in the levels of Aβ40 and Aβ42, neither in the soluble pool (D), nor in the membrane fraction (E) between SST-scFv8D3 treated group and the controls. Results are presented as mean ±SD. One-way ANOVA followed by Tukey's post hoc test (A) and unpaired t-test (C-E) were applied to measure the presence of statistically significant differences in the results. A significant p-value is defined as: >0.5 (ns), <0.05 (*), <0.01 (**), <0.005 (***).

The previous experiments confirmed that SST-scFv8D3 can activate neprilysin *in vitro* (Figure 2) and that the drug can be delivered into the brain when peripherally administered *in vivo* (Figure 3). Next, we investigated whether SST-scFv8D3 was also capable of increasing the concentration of neprilysin in the brain when injected intravenously at therapeutic doses and if elevated levels of neprilysin in the brain can subsequently reduce the levels of Aβ. For these experiments, APPswe mice were chosen over other AD mouse models based on a previous study demonstrating the resistance of Aβ to proteolytic degradation by neprilysin in Dutch, Flemish, Italian and Arctic mutations 54. Neprilysin protein levels in these mice (at eight and fourteen months) is significantly decreased compared to the wild-type mice (Figure 4A). In addition, we could detect an age-dependent decline in neprilysin expression in APPswe mice (Figure 4A), which is probably related to the increase in the formation and aggregation of Aβ. In this first therapeutic study, we measured the levels of neprilysin and Aβ in the brains of 14-month old APPswe mice after a single intravenous injection of 2 mg/kg of SST-scFv8D3 (Figure 4B). SST peptide alone without scFv8D3 was not used based on previous studies demonstrating its extremely short half-life of less than three min 48,55 and that analogs of SST with longer half-lives have been analyzed to see if they passed the BBB and their uptake in brain was low 56. Importantly, these analogs of SST have no affinity to the receptor subtype 4 57-59, which has high hippocampal and limited peripheral expression 36 and has been identified as the major regulator of neprilysin activity in the brain 60. A single injection of SST-scFv8D3 induced a significant increase in the levels of neprilysin in the membrane-bound fraction of the brain (Figure 4C). The concentration of neprilysin in the soluble pool of the brain was too low to be detected by our ELISA. No difference could be detected in the brain levels of Aβ40 and Aβ42, neither in the soluble pool (Figure 4D) nor in the membrane fraction (Figure 4E) between the SST-scFv8D3 treated group and the PBS injected controls.

### Three intravenous injections of SST-scFv8D3 increase neprilysin and decrease Aβ42 levels in the hippocampal area

**Figure 5:**
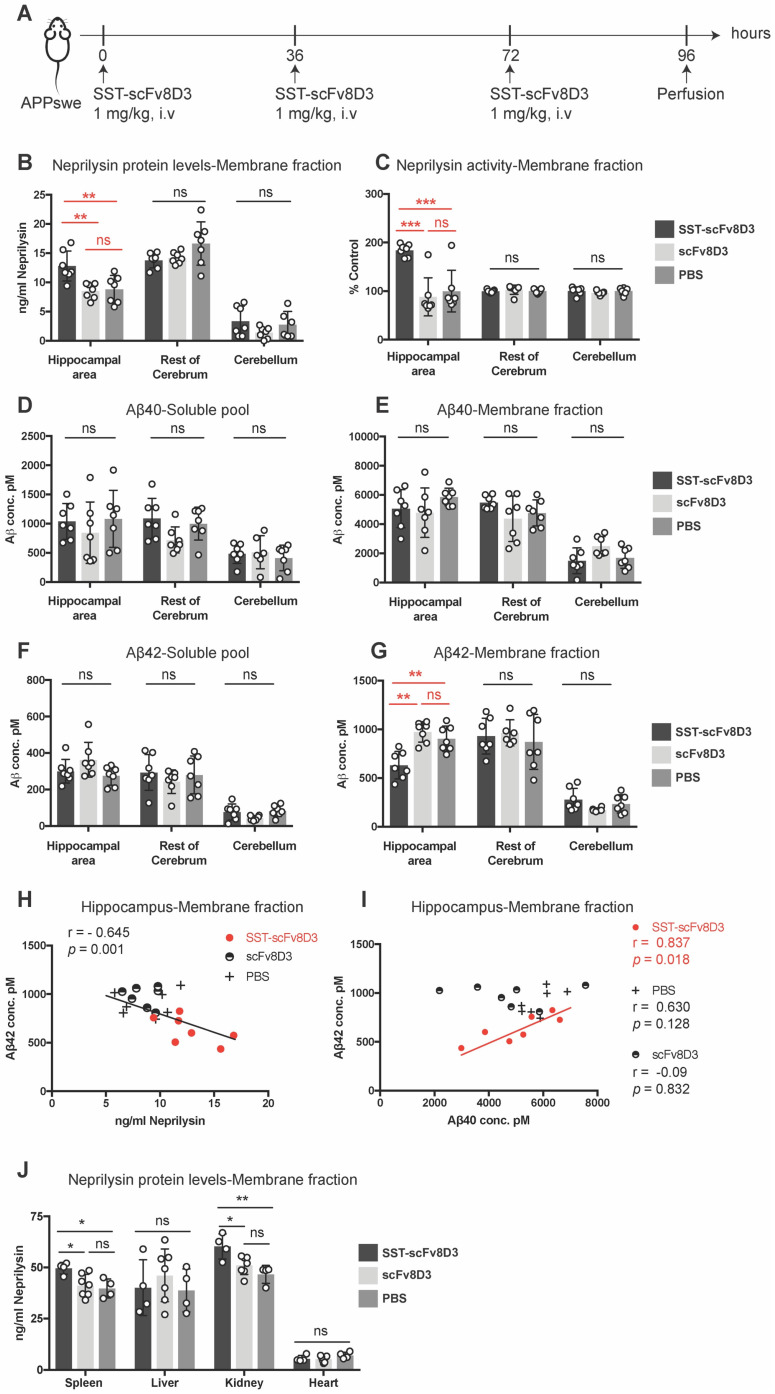
** Three intravenous injections of 1 mg/kg SST-scFv8D3 increase neprilysin levels and decrease Aβ42 in the hippocampal area.** (A) Schematic representation of the therapeutic study with repeated injections of SST-scFv8D3. Eight-month old APPswe mice were injected intravenously with 1 mg/kg of SST-scFv8D3 (n=7), an equimolar dose of scFv8D3 (n=7) or PBS (n=7) every 36 h (in total three injections). The mice were euthanized 24 h after the last injection and the brains dissected into three parts: Hippocampal area, rest of cerebrum and cerebellum. (B) A significant increase could be detected in neprilysin concentrations in the membrane fraction of hippocampus of the SST-scFv8D3 treated group compared to the controls measured with sandwich ELISA. No significant differences were detected in the rest of the cerebrum or the cerebellum. (C) A significant increase could be detected in neprilysin activity in the membrane fraction of the SST-scFv8D3 treated group compared to the controls. The differences were specific to the hippocampal area. No differences were detected in Aβ40 levels in the soluble pool (D) and membrane fraction (E), or in Aβ42 levels in the soluble pool (F) between SST-scFv8D3 treated group and the two control groups. (G) A significant decrease in Aβ42 levels detected in the membrane fraction of hippocampus in the treatment group. (H). A significant inverse correlation between the levels of neprilysin and Aβ42 in the membrane fraction of the hippocampus was obtained with Pearson's correlation. (I) A significant positive correlation between the concentration of Aβ40 and Aβ42 in the membrane fraction of the hippocampus of the SST-scFv8D3 treated mice only, obtained with Pearson's correlation. (J) A significant increase could be detected in neprilysin concentration in spleen and kidneys of the SST-scFv8D3 treated group (n=4) compared to scFv8D3 (n=7) and PBS (n=4) groups. No differences detected in liver and heart. Results are generated from two repetitive experiments, where four mice were used in first round and three mice in the second round. Results are presented as mean ±SD. One-way ANOVA was used followed by Tukey's post hoc test. A significant p-value is defined as: >0.5 (ns), <0.05 (*), <0.01 (**), <0.005 (***).

As a single injection of SST-scFv8D3 did not decrease brain Aβ despite increased neprilysin concentrations, we investigated whether three injections of SST-scFv8D3 could reduce Aβ levels. It has been previously demonstrated that SST mediated activation of neprilysin reaches the peak after 24 h and lasts for 36 h 28. Since we know that the brain uptake using the scFv8D3 transporter is fast (less than 2 h), we chose to do multiple injections of SST-scFv8D3 every 36 h to optimize the chance to detect differences in the levels of Aβ mediated by neprilysin. Younger (8 months old) APPswe mice were used, in which the total Aβ level is elevated compared to wild type mice, but aggregates and plaques are not formed yet as they are in 14 month-old mice, which we used in the single injection study. Neprilysin is most efficient in degrading monomers and small aggregates 61, hence, younger mice are likely to have less background signals from the large aggregates. In this experiment, a dose of 1 mg/kg of SST-scFv8D3 was given every 36 h (three injections in total), using scFv8D3 and PBS as negative controls (Figure 5A). The brains were perfused 24 h after the last injection and were dissected into three parts: hippocampus and the adjacent posterior parietal association area of the cortex (referred to as hippocampal area throughout the manuscript), rest of cerebrum and cerebellum. Similarly to the effect of a single injection, the triple injection of SST-scFv8D3 significantly elevated the concentration of neprilysin in the membrane fraction of the hippocampal area (Figure 5B). No significant differences between treated and untreated mice were observed in the neprilysin concentrations in the rest of the cerebrum or in the cerebellum (Figure 5B). Similar to the increase in protein expression, the activity of neprilysin was significantly enhanced in the hippocampus of the SST-scFv8D3 treated group (Figure 5C). Notably, the administration of SST-scFv8D3 resulted in a statistically significant decrease in the concentration of Aβ42 in the membrane fraction (Figure 5G). This decrease was specific to the Aβ42 levels in the hippocampal area. Importantly, the increase in neprilysin levels in the membrane fraction of the hippocampus significantly correlated with the decrease in Aβ42 levels (Figure 5H). A correlation between the Aβ40 and the Aβ42 levels in the membrane fraction of the hippocampal area showed a clear and significant correlation only in the treatment group (Figure 5I). No other brain region, neither in the soluble pool (Figure 5D) nor in the membrane fraction (Figure 5E) exhibited significant changes in the levels of Aβ40 or Aβ42 (Figure 5F). In addition to Aβ, neuroinflammation plays an integral role in AD initiation and progression 62. For this purpose, the hippocampus and the rest of cerebrum from the treated and untreated APPswe mice were screened for cytokines using a pro-inflammatory cytokine multiplex assay (MSD K15048D). No significant differences could be detected in the concentrations of IL-1b, IL-5, IL-6 and IL-12 between the groups (Figure S2). In addition to the brain, neprilysin levels were also quantified in spleen, liver, kidney and heart. Mice treated with SST-scFv8D3 exhibited a significant increase in neprilysin expression in spleen and kidneys, but no significant differences were detected in liver and heart among the groups (Figure 5J).

## Discussion

The current study aimed at finding a protein drug which enhances the extracellular degradation of Aβ, to potentially offer a new therapeutic option for AD. Neprilysin is a key player in the degradation of Aβ 51,63,64 and the neuropeptide SST enhances neprilysin activity. We therefore aimed to create a BBB penetrating SST and test it as a therapeutic strategy to treat AD.

The first part of the current study focused on assessing the ability of the recombinantly produced SST linked to a BBB transporter, SST-scFv8D3, to increase the activity of neprilysin. The interaction between SST receptors and SST has earlier been reported to be primarily through the SST amino acids at position 7 to 10 (phenylalanine, tryptophan, lysine, and threonine) 65, which are preserved in our designed SST-scFv8D3. However, adding a sterically large protein domain to a peptide can change its properties due to other reasons. Despite the fusion of the scFv8D3 to the N-terminal end of SST, the fusion protein was still capable of activating neprilysin. Our results confirmed that the N-terminal is not essential for SST ability to enhance neprilysin activity, justifying the addition of a BBB transporter at this position as a suitable strategy to create a SST peptide variant that can enter the brain.

The plasma half-life of SST peptide is just 2 to 3 min, severely limiting its potential clinical applications 48,66, as it is likely that such a drug would be cleared from the blood before reaching the brain. By adding the BBB transporter to SST, we hypothesized that we would also increase the half-life of the SST peptide due to the increased size. The scFv8D3 is 25 kDa and the SST peptide is 1.7 kDa. Binding of scFv8D3 to TfR located on the red blood cells 67 will also prolong the half-life of the formed fusion protein, as we have reported earlier 43,44. The half-life was extended 120 times (to 6 h) (Figure 3A), which is longer than that of other scFv proteins that have been reported to be around 1 h 68.

The BBB transporter and the half-life extension enabled us to achieve a high brain concentration of the drug. At 2 h post injection, the brain uptake of SST-scFv8D3 was similar to that obtained with another bispecific construct with the same transporter, di-scFv3D6-8D3 44. The plasma half-life of SST-scFv8D3 was around 6 h, but the brain retention of the therapeutic drug, as shown with the brain-blood ratio was even longer (Figure 3C). The 24 h post injection ratio demonstrated that SST-scFv8D3 stays for a longer time in the brain, where it binds to its targets (SST receptors), than it does in the blood. It is likely that the retention in the areas of the brain where SST receptors are present is even longer.

The brain uptake and biodistribution analysis were performed with [^125^I]-labelled SST-scFv8D3, which could slightly affect the pharmacokinetic properties compared to the non-labelled construct that was used for the therapeutic studies.

In the current study, we aimed to design a drug based on the neuropeptide SST that could be used to compensate for the low levels present in AD and in aging. We demonstrated that intravenous injections of SST-scFv8D3 could significantly increase the levels of neprilysin in the hippocampus of APPswe mice (Figure 5B). The effect was specific to the membrane fraction, where neprilysin is predominately located 22. The interaction between SST and SSTR promotes cell surface localization of neprilysin 28,52.

APPswe mice treated with SST-scFv8D3 also exhibited significantly decreased levels of membrane bound Aβ42 in the hippocampal region (Figure 5F). Not many studies have seen an effect on membrane bound Aβ42 since it is easier to reach the more soluble aggregates with antibodies. Targeting Aβ42 when it is membrane bound could have a stronger effect on cognition than targeting soluble Aβ42, due to the significance of membrane bound Aβ in triggering synaptic dysfunction and cell death in AD. This effect is mediated through the interaction with cell membrane components such as lipid rafts, leading to reduced membrane fluidity and formation of pores and ion-channel like structures 69,70. Thus, enhancing the degradation of membrane bound Aβ42 by our SST-scFv8D3 protein will hopefully prevent the pathological alteration in neuronal function that leads to cell death in AD.

SST-scFv8D3 effects were more pronounced on the aggregation prone Aβ42 isoform, but no significant decrease could be detected in Aβ40 levels. These findings are in agreement with (Saito et al; 2005) 28, where they demonstrated decreased Aβ42, but no significant decrease in Aβ40 concentration in primary neuronal cultures treated with SST. In the same study, an increase in Aβ42 concentration but not Aβ40 was observed in SST knockout mice. Our results are however in contrast to the findings demonstrating that neprilysin is equally efficient in degrading both Aβ40 and Aβ42 *in vitro*, or even better at degrading Aβ40 than Aβ42 71. Neprilysin has affinity for both Aβ40 and Aβ42 and has eleven cleavage sites on Aβ 72,73, indicating that enhanced activity of neprilysin could be followed by degradation of both Aβ40 and Aβ42. There is however a ten times higher concentration of Aβ40 in the membrane fraction *in vivo* compared to Aβ42 (Figure 5E-G). Therefore, to be able to detect significant differences in Aβ40 concentration, neprilysin needs to degrade more Aβ40 in total numbers, which could perhaps be achieved with longer treatment studies. *In vivo*, neprilysin has been displayed to degrade Aβ42 at the membrane more efficiently than Aβ40 51,74. In our study, a statistically significant correlation could be detected between the levels of Aβ42 and Aβ40 in the hippocampal membrane fraction of the treatment group, where low levels of Aβ42 were associated with relatively low levels of Aβ40 and high levels of neprilysin (Figure 5H-I). Reasons for why neprilysin degrades Aβ42 at the membrane faster than Aβ40 can be attributed to how these peptides and neprilysin interact at the membrane which might be different from their interaction in solutions. For instance, it has previously been reported that Aβ42 is more likely to adopt beta structure in association with membranes than Aβ40 75. It could also be that the localization of these two isoforms is different close to the cell membrane, making the interaction between neprilysin and Aβ42 more frequent.

SST acts on five different SST receptors, with high expression of receptor subtype-1 to 4 in the brain. They are found in a network of SST reactive neuronal cells including for instance the neocortex, amygdala and hippocampus 32. The location of these receptor subtypes in the brain correlates very well with the parts of the brain that are most severely affected by the aggregation of Aβ. The levels of SST in these areas have been found to be decreased during AD, potentially due to the early death of somatostatinergic interneurons 36, which is also associated with decreased neprilysin expression in the same areas 24. These findings are in accordance with previous studies showing that SST knock out mice have low neprilysin activity in the hippocampus, but not in the cerebellum 28. We could also confirm that the neprilysin levels were lower in the APPswe mice with AD symptoms (Figure 4A). Together, this indicates that the levels of neprilysin are not regulated by SST in the same way in the whole brain.

A recent publication using the rat TfR binder OX26 connected to neprilysin therapeutically caused a reduction in Aβ40 levels both in the CSF and brain parenchyma 76. However, the mentioned study was performed on wildtype rats and no AD model was used to test the therapeutic effects of the construct. Neprilysin is a promiscuous protease that degrades several other peptides including neuropeptide Y and enkephalin, which are involved in processes such as blood pressure regulation and nociception and hence such an approach is likely to be associated with more adverse effects compared to our design and that the drug will be active on more places than the indirect action of SST. Activation of neprilysin by SST-scFv8D3 will be limited to areas where both SSTR and neprilysin are highly expressed. This will lower the risk of side effects in areas where SSTR are not highly expressed. Importantly, in our study, no neprilysin activation was detected in the heart after treatment with SST-scFv8D3 (Figure 5J), which can have the potential in reducing the risk of severe adverse events such as the risk for heart failure, that today is often treated with neprilysin inhibitors 77. However, the side effects need to be further investigated, including effects in the gastro-intestinal tract, which expresses several SSTR subtypes 31. The effect and potential side effects of SST-scFv8D3 will be clarified upon identification of the SSTR subtypes involved in neprilysin regulation. Furthermore, no changes in the expression of pro-inflammatory cytokines were found in the brain of eight-months APPswe mice after treatment with SST-scFv8D3. More highlighted neuroinflammatory conditions and longer therapeutic studies might be required to display some anti-inflammatory effects of SST-scFv8D3.

Small molecule SST receptor agonists aiming at enhancing the clearance of Aβ through neprilysin activation have been proposed as therapeutic options in AD. However, they were administered either intracerebroventricularly to bypass the BBB 45,46,78 or as a chronic intraperitoneal administration on daily basis over a period of 28 days 79. These alternative administration strategies produced limited effects, where one dose gave an effect, but a higher dose caused an increase in Aβ aggregates, limiting their clinical application. In addition, a recent study displayed no Aβ lowering effects of the SSTR4 agonist NNC 26-9110 when administered intraperitoneally to a transgenic mouse model of AD over a period of two weeks 60. If one compares these results to the significant reduction of Aβ42 with our SST-scFv8D3 protein over a period of only four days, it further demonstrates the potency of our approach.

The most promising treatment strategies in clinical trials for AD aim at targeting the aggregated Aβ protofibrils 19,80. It has been demonstrated to be very difficult to create a binder that detects low molecular weight oligomers despite that the oligomers have been shown to be one of the most neurotoxic forms of Aβ aggregates 6,9. Thus, treatment strategies currently in clinical trials will not remove all neurotoxic species of Aβ and this might be the reason why they have very limited effect on cognition, since the nerve cells can continue to die. The aim of SST-scFv8D3 therapy is to pre-empt the formation of aggregates, providing a higher potential to completely halt disease progression, rather than targeting the already formed aggregates.

In conclusion, our findings may indicate that membrane-bound neprilysin preferentially degrades Aβ42. Upon ageing and onset of AD, the expression of neprilysin decreases, which could partially explain the observed accumulation of the aggregation prone Aβ42 in AD. With intravenous injections of our BBB permeable SST peptide, SST-scFv8D3, we were able to significantly increase the levels of membrane bound neprilysin, an effect that was followed by a significant and selective degradation of membrane bound Aβ42. The effects were also selective to the hippocampal region, an area that is severely affected in AD pathology. Taken together, if used early in disease progression, SST-scFv8D3 may serve as a possible therapeutic option in AD, with the potential to better prevent neurotoxicity than the drugs currently being tested in clinical trials. The aim with SST-scFv8D3 is to degrade the monomeric and small species of Aβ, thus, preventing the aggregates from being formed, an event that takes place much earlier that the occurrence of learning and memory impairment. Further and longer-term studies can focus on investigating the effects of SST-scFv8D3 in regulating cognitive and learning functions. In the future, it would also be possible to combine SST-scFv8D3 treatment with other brain penetrating therapeutic approaches, such as bispecific monoclonal antibodies against the larger aggregates of Aβ, providing a more multifaceted treatment strategy.

## Materials and methods

### Cloning of SST-scFv8D3

A scFv consisting of the heavy and light variable domains of the 8D3 antibody 81 was linked to the N-terminal end of the 14 amino acid SST peptide (AGCKNFFWKTFTSC) through an in-house designed linker (APGSYTGSAPG) 43. The two variable regions of 8D3 were connected to each other through a glycine-serine (G4S)3 linker 82. To the N-terminal end of scFv8D3, a poly-6 His-tag (HHHHHH) was added using the same (APGSYTGSAPG) linker. The whole construct was cloned into pcDNA3.4 vector (GeneArt) with a signal peptide on the N-terminal end.

### Recombinant expression and purification

The recombinant SST-scFv8D3 protein was transiently expressed, as previously described 47. Briefly, human Expi293 cells were transfected with the pcDNA3.4 vector containing SST-scFv8D3 gene using polyethylenimine (PEI) (Polysciences 24765-1) as a transfection reagent and valproic acid (VPA) (Sigma P4543) as a cell cycle inhibitor. Nine days after transfection, the cells were harvested by centrifugation at 2000 x g and the supernatant was filtered using low protein binding 0.22 μm filter units (Corning 430513). The protein was purified using an ÄKTA start system with nickel column (HisTrap™ Excel, GE17-3712-06, GE Healthcare). Imidazole (Merck 1047160250) at a concentration of 500 mM in phosphate buffer was used for elution. Directly after elution, the fractions were concentrated and the buffer was exchanged to PBS. The concentration of the purified protein was measured using absorbance at 280 nanometer (nm).

### In vitro analysis of SST-scFv8D3

The eluted protein was mixed with LDS sample buffer (ThermoFisher B0007) and loaded onto 4-12% Bis-Tris protein gels (ThermoFisher NW04125) without adding reducing agents. A pre-stained protein marker (ThermoFisher 26616) was used as a molecular weight standard. The gel was stained with PAGE blue protein solution (ThermoFisher 24620) to confirm the size and purity of the purified protein. Binding of the purified SST-scFv8D3 to TfR was assessed with a previously described ELISA 50, using the bispecific antibody RmAb158-scFv8D3 43 as a positive control and SST peptide (without the additional scFv8D3) as a negative control. Ninety-six wells half area plates (Corning Incorporated 3960) were each coated with 50 ng of the recombinant mouse TfR protein in PBS. After blocking for 2 h at room temperature (RT) with 1% BSA in PBS, serial dilutions of SST, SST-scFv8D3 and RmAb158-scFv8D3 were added and incubated for 2 h at RT while shaking. The rat anti-somatostatin antibody clone Y7 (Merck mAb354) was added only to the wells incubated with SST and SST-scFv8D3 for 2 h at RT. For the detection, horse-radish peroxidase (HRP) conjugated secondary antibodies goat anti-mouse (for RmAb158-scFv8D3 incubated wells) (Sigma 12349) and goat anti-rat (for SST and SST-scFv8D3 incubated wells) (Sigma AP136P) were used, followed by signal development with K-blue aqueous TMB (Neogen Corp 331177). The absorbance was measured at 450 nm using FLUOstar Omega microplate reader (BMG Labtech). All dilution series (except the coated protein) were made in ELISA incubation buffer (1x PBS with 0.1% BSA and 0.05% Tween-20), and the wells were washed between each step with ELISA washing buffer (1x PBS with 0.05% Tween-20).

### Cell culture and primary neurons

Human neuroblastoma cell lines SHSY5Y were grown at 37 ^o^C with 5 % CO_2_ in a 1:1 mixture of minimum essential medium MEM (Gibco 41090036) and Ham's F-12 medium (Gibco 21127002), supplemented with 10% fetal bovine serum (Gibco 10270106). The cells were plated in 96 well black plates with clear bottoms (Corning Incorporated 3603) at a density of 5 000 cells per well. Primary Hippocampal/Striatal neurons were isolated from C57Bl/6 mice at embryonic day 14. The embryonic brain tissue was dissected in Hank's buffered salt solution (HBSS, Invitrogen 88284) containing 50 U/ml penicillin and 50 mg/ml streptomycin (Thermofisher 15140122) and 8 mM Hepes buffer (Invitrogen 15630-080). Following centrifugation for 3 min at 150 x g, the pellet was resuspended in HBSS and any remaining blood vessels were let to sediment for 15 min at RT. The supernatant was centrifuged for 5 min at 150 x g and the cell pellet was resuspended in neurobasal medium (Invitrogen 21103049) supplemented with 1x B-27 (Invitrogen 17504001), 50 U/ml penicillin, 50 mg/ml streptomycin (Thermofisher 15140122) and 20 mM L-glutamine (Invitrogen 25030081). The cells were plated in 96 well black plates with clear bottoms (Corning Incorporated 3603) at a density of 10 000 cells per well. The media was changed completely the day after seeding and half of the media was changed every 2-4 days after that. All the procedures with primary neurons were conducted according to the Swedish ethical policies regarding animal experiments and were approved by the Uppsala County Animal Ethics Board (#5.8.18-08472/18).

### Neprilysin activity measurement in vitro

Activity of cell-surface neprilysin in primary neurons and SHSY5Y cells was measured as described previously 52. To eliminate the effect of the serum, the SHSY5Y cells were starved 48 h prior to the addition of the proteins by changing to a serum free medium. The primary neurons were treated with 1 μM of SST peptide (PeptaNova 4032), SST-scFv8D3 or scFv8D3 for 24 h at 37 ºC. The same incubation time was used for SHSY5Y cells, with the exception that a higher amount (10 μM) of proteins was used. Cyclo-SST (Tocris 3493) was used as SSTR antagonist. After the incubation, the media was removed and the cells were washed with 0.1 M MES (pH 7.0). The cells were then incubated for 1 h at 37 ºC with a substrate mix consisting of 0.2 M MES, EDTA-free complete protease inhibitor (Roche 05056489001), Z-LLLaldehyde (final concentration of 1 μM, Peptide Institute 3175) and Suc-Ala-Ala-Phe-MCA (Final concentration of 0.5 mM, Sigma S8758) in assay buffer (50 mM Tris-HCl pH 7.6, 25 mM NaCl, 5 mM ZnCl2). The neprilysin inhibitor, thiorphan (final concentration 10 μM, Sigma T6031) diluted in assay buffer (50 mM Tris-HCl pH 7.6, 25 mM NaCl, 5 mM ZnCl2), was added to half of the wells. Following this, phosphormamidon (final concentration 0.1 mM, PeptaNova 4052-25) and Leucine aminopeptidase (final concentration 0.1 mg/ml, Sigma L5006) were added to the plates and further incubated for 30 min at 37 ºC. Fluorescence intensity was measured at excitation 320 nm and emission 410 nm using FLUOstar Omega microplate reader (BMG Labtech). Due to limitations in the plate reader, it was not possible to adjust the wavelengths to those best used for the detection of MCA fluorescence (excitation 380 nm and emission at 460 nm), which might affect the sensitivity of the generated fluorescent readings. The difference in fluorescence intensity between the thiorphan-negative and thiorphan-positive reactions defined neprilysin activity.

### Animals

The APPswe transgenic mouse model of Alzheimer's disease, harboring the Swedish mutation (AβPP KM670/671NL), maintained on a C57Bl/6 background was used in this study. Both male and female animals were used, and littermates served as a wild-type control. The mice were housed in an animal facility at Uppsala University, with free access to water and food under controlled temperature and humidity. All procedures were conducted according to the Swedish ethical policies regarding animal experiments and approved by the Uppsala County Animal Ethics Board (#5.8.18-13350/17).

### Radiolabeling of SST-scFv8D3 with iodine-125

The recombinant SST-scFv8D3 was labelled with iodine-125 using Chloramine-T as described previously 83. Briefly, 650 nM of SST-scFv8D3 (Mw 31 kDa) was mixed with the iodine-125 stock solution (NEZ033A010MC, Perkin Elmer Inc, Waltham) and 1 mg/ml of Chloramine-T (Sigma 857319) in PBS. The reaction was stopped after 90 s with 1 mg/ml Sodium meta-bisulphite (Sigma 08982). The radio-labelled SST-scFv8D3 was purified from free and unbound iodine and eluted in PBS using NAP columns (VWR 17-0853-02). The labelling procedure was performed just 1 h before the *ex-vivo* study.

### Ex-vivo study

Brain uptake, peripheral biodistribution and plasma half-life of SST-scFv8D3 was investigated in C57Bl/6 wild-type mice (7-8 months-old). Mice (n=9) were intravenously injected via the tail vein with 0.44±0.08 MBq of [^125^I]SST-scFv8D3. Eight-microliter blood samples from the tail vein were obtained at 0.5, 1, 2, 4, 6 and 24-h post injection. The mice were euthanized 2 h (n=3), 6 h (n=3) and 24 h (n=3) post injection by transcardial perfusion with 0.9% physiological saline. Brains were dissected, divided into two hemispheres, snap frozen on dry ice and subsequently stored in -80 °C until further analysis. Lung, liver, kidney, spleen, heart, muscle, bone, pancreas, skull and thyroid were also isolated. Radioactivity in brain, peripheral organs and blood was measured using a χ-counter (1480 Wizard, Wallac Oy, Turku, Finland). Data are presented as percentage of injected dose per gram tissue (% ID/g).

### SST-scFv8D3 treatment study-1

The effect of the recombinantly produced SST-scFv8D3 protein on neprilysin and Aβ levels in the brain was investigated in 14-month old APPswe transgenic mice (n=8). The mice were divided into two groups (n=4/group), where one group was given a single intravenous injection of SST-scFv8D3 (2 mg/kg) and the second group received a single intravenous injection of PBS. The mice were euthanized 24 h post injection by transcardial perfusion with 0.9% physiological saline. Brains were isolated and homogenized with Tris-buffered saline at a 1:5 weight/volume ratio, containing cOmplete^TM^ protease inhibitors (Roche 05056489001). The homogenates were then centrifuged for 1 h at 16 000 x g at 4 ºC and the supernatant was collected. This fraction contained extracts of soluble proteins; thus, it was termed as the soluble pool throughout the study. The remaining pellet was resuspended at 1:5 weight/volume ratio with Tris-buffered saline containing 1% Triton-X, followed by centrifugation for 1 h at 16,000 x g at 4 ºC. The resulting supernatant containing extracts of soluble membrane bound proteins was collected. This fraction is therefore termed as the membrane fraction throughout the study. The obtained fractions were aliquoted and stored at -80 ºC.

### SST-scFv8D3 treatment study-2

Based on the results from the first treatment study, a second study was performed that included three injections of SST-scFv8D3. In this second study, we altered the dose of the drug from 2 mg/kg to 1 mg/kg. It has been previously reported that TfR uptake is better when protein-based drugs are given at tracer dose compared to therapeutic doses. Doses above 1 mg/kg of the bispecific RmAb158-scFv8D3 seems to saturate the transcytosis of TfR, leading to reduced brain uptake 84. To this end, eight month old APPswe transgenic mice were divided into three groups (n=7/group) where one group was given an intravenous injection of SST-scFv8D3 (1 mg/kg, corresponding to 32 nmol/kg, Mw 31 kDa) every 36 h. The second group received an intravenous injection of scFv8D3 (0.9 mg/kg, corresponding to 32 nmol/kg, Mw 28 kDa) every 36 h. The third group was administered an intravenous injection of PBS, at the same time points as the first and second groups. The mice were euthanized 24 h after the third injection by transcardial perfusion with 0.9% physiological saline, followed by dissection of the brain. The cerebellum was separated from the rest of the brain, and the hippocampus with the adjacent posterior parietal association area of the cortex (referred to as hippocampal area throughout the manuscript) was dissected from the rest of the cerebrum. In addition, spleen, kidney, liver and heart were also isolated. The three brain parts (hippocampal area, rest of the cerebrum and cerebellum) and the peripheral organs were homogenized and processed to obtain the soluble and membrane bound extracts as described in the previous section.

### Immunohistochemistry

To check regional distribution of SST-scFv8D3 in the brain, His-tag staining was performed on brain sections prepared from the right hemispheres of the injected C57Bl/6 mice. Sagittal cryo-sections 50 μm thick were prepared and fixated for 30 min with cold acetone. Endogenous peroxidase activity was blocked with 0.3% H_2_O_2_ (Chem-supply 426001000) for 30 min at RT. After rinsing with 1x PBS, the sections were incubated for 1 h at RT with 2.5% normal goat serum (Victor Laboratories S1012), followed by an additional 1 h incubation with HRP-conjugated anti-6x His antibody (abcam 1187). After rinsing the slides three times with 1 x PBS, the signal was developed using ImmPACT DAB substrate (Vector Laboratories SK-4105). For neprilysin staining, formalin-fixed paraffin embedded 4 μm thick coronal brain sections from 3-month old C57Bl/6 mice were prepared. The sections were autoclaved for 5 min for antigen retrieval and immunostained with an antibody against neprilysin (56C6, Novocastra, NCL-L-CD10-270) as described previously 24. The slides were mounted and images were acquired using Zeiss Axio Observer Z1.

### Neprilysin activity measurement in vivo

Activity of neprilysin in the membrane fractions was measured as described previously 85. Ninety-six well half area black plates (Corning Incorporated 3694) were coated overnight with 1.6 μg/ml of the capture antibody (Goat-anti mouse neprilysin) (R&D DY1126) in PBS at 4 ºC. After blocking the plates with 1% BSA in 1 x PBS for 2 h at RT, membrane fractions were diluted in assay buffer (50 mM Tris-HCl pH 7.6, 25 mM NaCl, 5 mM ZnCl2) and incubated for 2 h at RT. The neprilysin inhibitor, thiorphan (final concentration 10 μM, Sigma T6031), was added to half of the wells. Fluorogenic substrate (MCA, final concentration 10 μM, R&D, ES005) diluted in assay buffer (50 mM Tris-HCl pH 7.6, 25 mM NaCl, 5 mM ZnCl2) was added and the plates were incubated for an additional 2 h at 37 ºC in the dark. Fluorescence intensity was measured at excitation 320 nm and emission 410 nm using FLUOstar Omega microplate reader (BMG Labtech). The difference in fluorescence intensity between the thiorphan-negative and thiorphan-positive reactions defines neprilysin activity.

### Neprilysin protein-levels ELISA

Levels of neprilysin in the membrane fractions (TBS-Triton soluble fractions) were measured using neprilysin ELISA according to the manufacturers protocol (R&D DY1126). Ninety-six well half area plates (Corning Incorporated 3960) were coated overnight with 1.6 μg/ml of the capture antibody (Goat-anti mouse neprilysin) in PBS at 4 ºC. The following day, the plates were blocked with 1% BSA in 1 x PBS for 2 h at RT. Membrane fractions were diluted 1:10 (v/v) in ELISA incubation buffer (1 x PBS with 0.1% BSA and 0.05% Tween-20) and incubated for 2 h at RT. Biotinylated goat anti-mouse neprilysin detection antibody (400 ng/ml) was added and the plates were further incubated for additional 2 h at RT. This was followed by incubation with Streptavidin-HRP (diluted 1:4000 v/v) (Mabtech 3310-9-1000) for 1 h at RT. The signal was developed using K-blue aqueous TMB (Neogen Corp) and absorbance was measured at 450 nm using FLUOstar Omega microplate reader (BMG Labtech).

### Aβ ELISA

Levels of Aβ40 and Aβ42 in the soluble pool and membrane fraction of the APPswe mice were measured using Aβ ELISA as described previously 17. In this sandwich ELISA setup, a capture antibody directed towards the C-terminal end and a detection antibody directed towards the N-terminal end of Aβ were used. Such a setup will only detect the full-length Aβ peptides, eliminating the possibility of detecting products generated by the degradation of Aβ by neprilysin. To this end, ninety-six well half area plates (Corning Incorporated 3960) were coated overnight at 4 ºC with C-terminal specific Aβ40 (Invitrogen 44-136) or Aβ42 rabbit antibodies (Invitrogen 700254), followed by blocking with 1% BSA in 1x PBS for 3 h at RT. Extracts from both the soluble pool and membrane fractions of the brain were diluted 1:10 (v/v) in ELISA incubation buffer and incubated overnight at 4 ºC. Biotinylated 82E1 (IBL 10326) and Streptavidin-HRP were used for the detection. The signal was developed and absorbance was measured as described in the previous section.

### Statistical analyses

All data are presented as mean ± SD. Data were analyzed with one-way ANOVA followed by Dunnett's or Tukey's post hoc test or with unpaired t-test. Pearson's correlation was applied to investigate the correlation between neprilysin and Aβ levels in the membrane fraction of the hippocampus. Statistically significant differences were defined as: p-value >0.5 (ns), P-value <0.05 (*), P-value <0.01 (**), P-value <0.005 (***).

## Supplementary Material

Supplementary figures.

## Abbreviations

Aβ: amyloid-β
AβPP: amyloid-β precursor protein
AD: Alzheimer's disease
BBB: blood-brain barrier
CSF: cerebrospinal fluid
scFv: single chain fragment variable
SST: somatostatin
TfR: transferrin receptor
