# Supplementary material for: Enhanced neprilysin-mediated degradation of hippocampal Aβ42 with a somatostatin peptide that enters the brain
Source: Theranostics. 2021 Jan 1;11(2):789–804. doi: 10.7150/thno.50263 (PMC7738863; doi:10.7150/thno.50263)
Supplement: Supplementary file 1 — Supplementary figures. [file thnov11p0789s1.pdf]

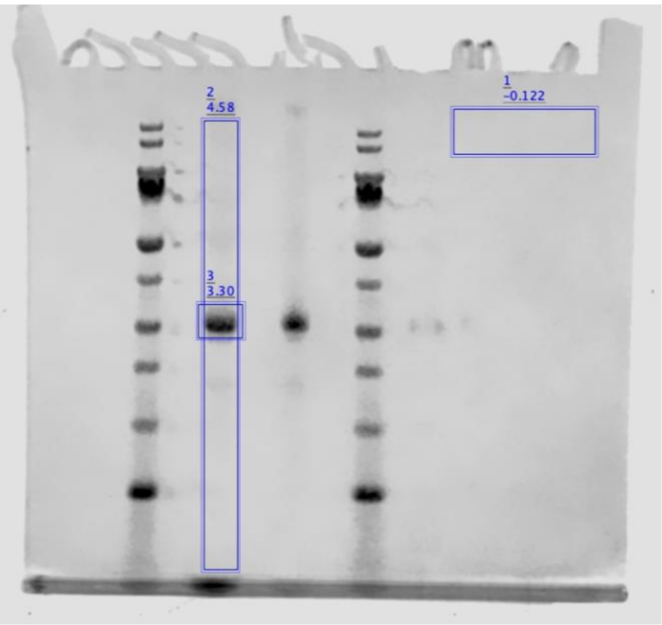

Figure S1 Estimation of SST-scFv8D3 purity with Image Studio software. SDS-PAGE of the purified SST-scFv8D3 from Figure 1B was analyzed using Image Studio software. Estimation was based on measuring the intensity of SST-scFv8D3 band (rectangle.3) from the intensity of the whole well (rectangle.2) after minimizing the background signal (rectangle.1). Purity of SST-scFv8D3 was estimated to be approximately 70%.

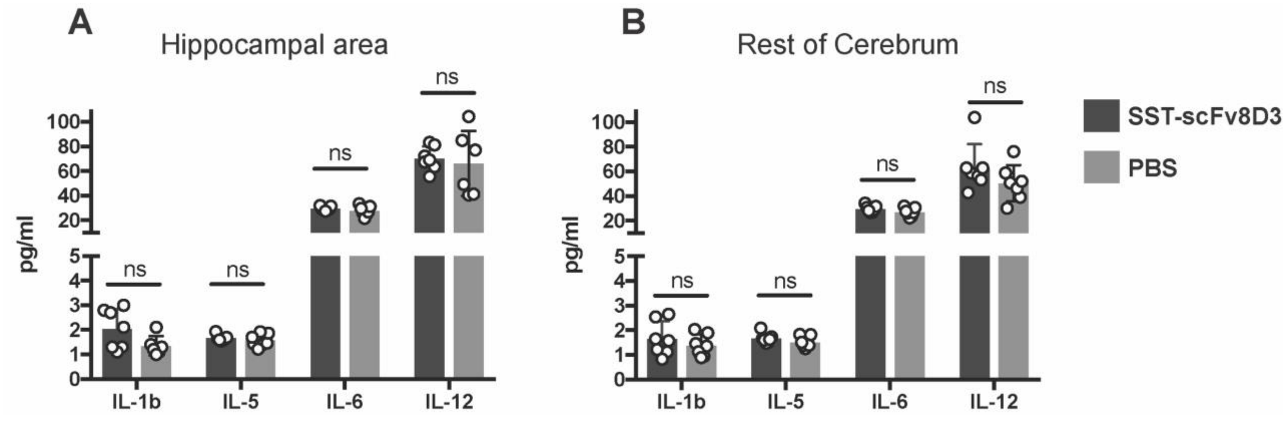

Figure S2 Concentration of cytokines in the brain of eight-months-old APPswe mice after three intravenous injections of 1 mg/kg SST-scFv8D3 using PBS as a control. No significant differences detected in the concentrations of IL-1b, IL-5, IL-6 and IL-12 between the groups, neither in the hippocampal area (A) nor in the rest of cerebrum (B). Measurements carried out using a pro-inflammatory cytokine multiplex assay (MSD K15048D). Results are presented as mean ±SD. Unpaired t-test was applied to measure the presence of statistically significant differences in the results. A significant p-value is defined as: >0.5 (ns), <0.05 (*), <0.01 (**), <0.005 (***).
